# LSD1: Expanding Functions in Stem Cells and Differentiation

**DOI:** 10.3390/cells10113252

**Published:** 2021-11-20

**Authors:** Carlos Martinez-Gamero, Sandhya Malla, Francesca Aguilo

**Affiliations:** 1 Department of Molecular Biology, Umeå University, SE-901 85 Umeå, Sweden; carlos.martinezgamero@unil.ch (C.M.-G.); sandhya.malla@umu.se (S.M.); 2 Wallenberg Centre for Molecular Medicine, Umeå University, SE-901 85 Umeå, Sweden

**Keywords:** LSD1, KDM1A, lysine-specific demethylase, epigenetics, histone methylation, non-histone substrate, embryonic stem cells, induced pluripotent stem cells, self-renewal, pluripotency, differentiation

## Abstract

Embryonic stem cells (ESCs) and induced pluripotent stem cells (iPSC) provide a powerful model system to uncover fundamental mechanisms that control cellular identity during mammalian development. Histone methylation governs gene expression programs that play a key role in the regulation of the balance between self-renewal and differentiation of ESCs. Lysine-specific demethylase 1 (LSD1, also known as KDM1A), the first identified histone lysine demethylase, demethylates H3K4me1/2 and H3K9me1/2 at target loci in a context-dependent manner. Moreover, it has also been shown to demethylate non-histone substrates playing a central role in the regulation of numerous cellular processes. In this review, we summarize current knowledge about LSD1 and the molecular mechanism by which LSD1 influences the stem cells state, including the regulatory circuitry underlying self-renewal and pluripotency.

## 1. Introduction

Epigenetic mechanisms such as DNA methylation, histone modifications, and non-coding RNA-mediated regulation, dictate chromatin architecture and therefore, tightly determine the spatial accessibility of transcription factors to genetic loci. Such mechanisms, referred to as the epigenome, are heritable, do not propagate in the DNA sequence, and are highly dynamic to provide cellular plasticity for cells to respond to environmental and developmental cues. There is an interplay amongst the different layers of epigenetic information which are constantly reshaped during early development and differentiation to regulate the transcriptional landscape.

Two copies of the core histones H2A, H2B, H3, and H4 assemble with 147 base pairs of DNAs to form the basic unit of the chromatin, the nucleosome. Dynamic and reversible post-translational modifications (PTMs) occurring at the N-terminal tails of histones are regulated by a repertoire of writers, erasers, and readers that add, remove and recognize the chemical mark. The histone code includes phosphorylation, acetylation, methylation, ubiquitination, sumoylation, and GlcNAcylation among others [1].

Amongst those chemical modifications, lysine methylation is a widespread PTM which involves the transfer of up to three methyl groups to the ε-amino group of a lysine (K) residue, resulting in either mono-, di-, or trimethyl lysine. One of the major features of histone lysine methylation is that its regulatory function depends on its position and methylation state [2]. For example, H3K4me3 is restricted to promoters of actively transcribed genes, whereas H3K27me3 is enriched within repressed regions, and covers the gene body and flanking regions. H3K4me1 alone is the hallmark of primed enhancers, and in combination with H3K27me3 or H3K27Ac is associated with poised and active enhancers, respectively. In embryonic stem cells (ESCs), many developmental genes harbor promoters with the H3K4me3 and the H3K27me3 marks. These bivalent domains lead to a poised transcriptional status and allow timely activation while maintaining repression in the absence of differentiation cues. In particular, H3K4me2 is associated with both active promoters and enhancers.

Histone methylation was thought to be static until the discovery of lysine-specific histone demethylase 1 (LSD1; also known as KIAA061/KDM1/AOF2/BHC110) [3], which demethylates H3K4me1/2 and H3K9me1/2 depending on the cellular context. Recently, a neuron-specific isoform of LSD1 (LSD1n) has been shown to demethylate H4K20me1/2 [4]. Additionally, in recent years, a plethora of non-histone substrates have been detected as targets of LSD1, revealing additional complexities in the role of this demethylase in multiple pathways triggered in diverse cellular processes, including embryonic development. ESCs are a suitable model for studying complex epigenetic mechanisms involved in development due to their intrinsic capacity to give rise to all the somatic cells of an organism. The regulation of the epigenetic landscape is fundamental in order to activate or silence specific gene expression programs that ensure proper differentiation [5]. LSD1, as a histone remodeler, plays a critical role in the regulation of such epigenetic landscape in ESC biology and in other relevant biological roles such as cancer [3,6,7]. In this review, we will focus on the function of LSD1 in ESC self-renewal, pluripotency and reprogramming. Within the same context, we will also discuss the crosstalk between histone methylation and other epigenetic marks.

## 2. Structure and Enzymatic Activity of LSD1

**Figure 1:**
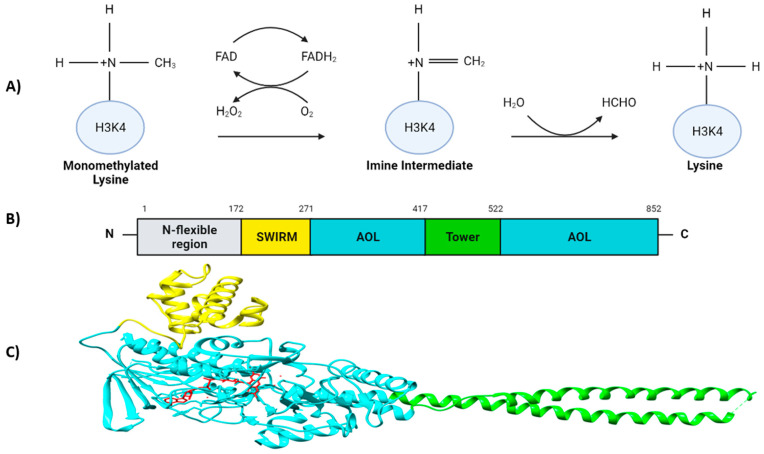
Overview of the demethylation process and the LSD1 structure. (**A**) LSD1-mediated demethylation of monomethylated H3K4. (**B**) Domain organization of human LSD1 depicting the N-flexible region (grey), the SWIRM domain (yellow), the AOL domains (cyan), and the Tower domain (green). (**C**) Overall structure of LSD1. The SWIRM (yellow), AOL (cyan) and Tower domain (green) are represented. The catalytic center of LSD1 located inside of the two AOL subdomains is colorized in red.

LSD1 is a flavin-adenine dinucleotide (FAD)-dependent amine oxidase (AO) that catalyzes the demethylation of mono- and di-methyl groups, but not tri-methyl groups, on H3K4 and H3K9, triggering transcriptional repression and activation, respectively [3,8]. Additionally, plenty of non-histone targets have also been identified [9,10]. During the demethylation reaction, the methyl-lysine group is oxidized by FAD, forming an imine intermediate which is consequently hydrolyzed into formaldehyde. Thereafter, the reduced FADH_2_ is again re-oxidized by an oxygen releasing hydrogen peroxidase (Figure 1A) [3,11,12]. 

LSD1 is a highly conserved protein and is structurally divided into three major protein domains: (i) the N-flexible region; (ii) the SWI3/RSC8/MOIRA (SWIRM) domain and (iii) a C-terminal amino oxidase-like (AOL) domain which is divided into two fragments by the TOWER domain [13]. The N-flexible region has been shown to be dispensable for the LSD1 demethylase activity, but essential for LSD1 nuclear localization [13,14]. In contrast to other SWIRM domains, the SWIRM domain of LSD1 does not bind to DNA, but it contributes to maintain the protein stability of LSD1, and it serves as a docking site for interaction with other proteins [13,15,16,17]. The AOL domain is the catalytic region of LSD1. It is formed by two lobes; the first one structurally binds together with SWIRM domain which contains the FAD-binding site, and it is where the oxidation process occurs; the second one presents the substrate recognition site. Both lobes form a cavity where the demethylation activity is produced in the catalytic center [13]. The TOWER domain is a protruding structure from the AOL domain consisting of two α-helices [8,13]. It is connected to the catalytic site of LSD1 acting as a binding platform for its interaction partners such as the CoREST complex, which is crucial for its H3K4 demethylase activity [13,18,19] (Figure 1B,C). It has been recently discovered that the AOL domain, together with the CoREST complex, had an affinity to bind to extranucleosomal DNA [20,21].

LSD2 (KDM1B/AOF1) is a protein homolog of LSD1 [22]. LSD2 is also a FAD-dependent amino oxidase with specificity to only demethylate H3K4me1/2, but not other histone and non-histone substrates, except for some determined inflammatory promoter genes related with NF-κB proteins, where LSD2 demethylates H3K9me2 [22,23]. Structurally, although LSD2 also presents a SWIRM and an AOL domain, it shares less than 31% of the sequence similarity with LSD1. The main difference is that LSD2 lacks the protruding TOWER structure and the zinc-finger element located in the N-terminal, which is crucial for FAD binding and for its active conformation by interacting with the AOL domain [22,24,25]. Although both LSD1 and LSD2 present FAD-demethylation activity, they have different functions in the cell. Hence, whereas LSD1 can act either as a transcriptional activator or repressor, binding to promoters and gene enhancers [6,26], LSD2 preferentially associates with the coding region of transcriptionally activated genes. LSD2 interacts with elongation factors such as Pol II and Cyclin T1, and by affecting the methylation dynamics of these genes together with the methyltransferase NSD3, it positively regulates the elongation process of actively transcribed genes [22,27].

## 3. Regulation of Gene Expression Mediated by LSD1: Transcriptional Repression and Activation

**Figure 2:**
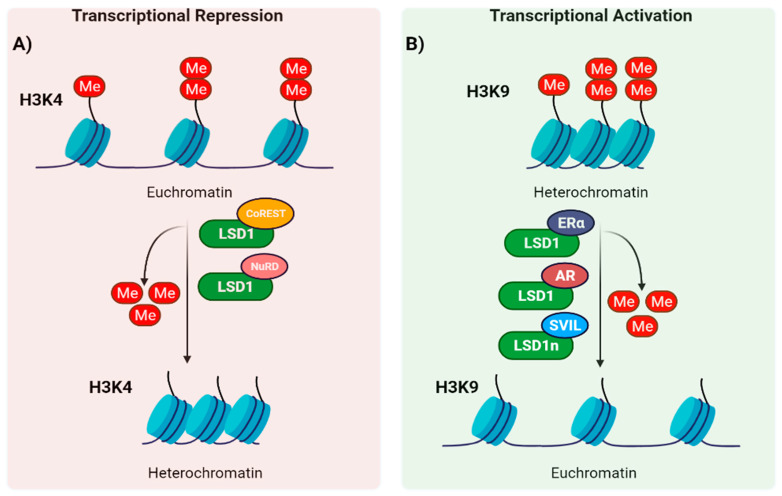
Transcriptional regulation of LSD1 by its demethylation activity on histone substrates. (**A**) LSD1 interacts with CoREST, NuRD and other protein complexes (not shown) to catalyze H3K4me1/me2 demethylation, resulting in transcriptional repression. (**B**) In some cellular contexts, LSD1 can interact with ERα and AR and activate transcription by demethylation of H3K9me2/me1. The isoform LSD1n presents affinity for H3K9 methyl groups through supervillin (SVIL) binding. LSD1-mediated methylation of H4K20me2 is not represented for simplicity.

LSD1 can form different protein complexes to shape the chromatin into a repressive or active configuration, depending on whether it demethylates H3K4 or H3K9. One of the best-characterized complexes in which LSD1 takes part is the CoREST transcription repressor complex, consisting of LSD1, RCOR1 (also known as CoREST), HDAC1, HDAC2, ZNF217, PHF21A and HMG20B [28,29,30,31]. Although LSD1 alone can demethylate histones or peptide substrates in vitro, the formation of the LSD1-RCOR1 complex is required for the LSD1 demethylase activity within the nucleosome [21,32]. In addition, such interaction is also required for LSD1 stability [19,33]. PHF21A binds unmethylated H3K4, the reaction product of LSD1, stabilizing LSD1 on its target regions and thereby mediating demethylation of the surrounding nucleosomes [34]. Therefore, recognition of the unmodified state on histone tails seems to be as crucial as post-translational modifications of histone for transcriptional regulation (Figure 2A).

Another well-studied complex important for transcriptional repression is the Mi-2/nucleosome remodeling and deacetylase (NuRD) complex. It is composed of, among others, the ATP-dependent chromodomain helicase DNA-binding protein (CHD) 3/4, HDAC1/2, metastasis-associated protein (MTA) 1/2/3, retinoblastoma binding protein (RBBP) 4/7 (also known as RbAp48/46), the methyl-CpG-binding domain protein (MBD) 2/3 and GATAD2A/GATAD2B [34]. The NuRD complex plays a pivotal role in cell signaling pathways, and it facilitates lineage commitment of ESCs by attenuating the expression of pluripotency genes, thus sensitizing cells to a loss of self-renewal factors [35]. In both the CoREST and the NuRD complexes, deacetylation of histone H3 mediated by HDAC1 and HDAC2 is combined with the demethylation of histone H3K4 in order to repress transcription (Figure 2A).

Although context-specific, it has been shown that LSD1 also serves as a transcriptional activator. Hence, LSD1 associates with androgen receptor (AR) and estrogen receptor (ER), and upon hormone treatment, they co-localize to promoters, resulting in selective H3K9 demethylation [36,37]. However, at the mechanistic level, the LSD1-histone peptide co-crystal structure seems not compatible with H3K9 demethylation (Figure 2B) [8,18].

Through RNA alternative splicing, combinatorial retention of exons E2a and E8a of LSD1 can be included in the mature mRNA, generating four possible isoforms (conventional LSD1, LSD1+2a, LSD1+8a, LSD1+2a+8a). Notably, whereas LSD1 and LSD1+2a are ubiquitously expressed, the expression of E8a-containing isoforms is restricted to the nervous system (referred to as LSD1n, neuronal form), and required for neuronal maturation [38]. It has been shown that LSD1n exhibits robust H3K9me2, but not H3K4me1/2, demethylase activity which is mediated through interaction with supervillin (SVIL) occurring through the exon 8a during neuronal differentiation [39]. Moreover, LSD1n also presented a new histone substrate specificity, targeting the repressive mark H4K20me2 by interacting with CREB and MEF2 [4]. MEF2 facilitated the binding of LSD1 to the enhancer sites of neuronal activity-regulated genes. Hence, LSD1n has been related to neuronal-specific gene expression together with long-term memory and spatial learning abilities [4]. Moreover, Toffolo et al., described that phosphorylation of the second residue coded by the exon 8a caused a local conformational change that led to detachment of the corepressors CoREST and HDAC1/2 from LSD1+8a [40]. Hence, this post-translational modification switched LSD1n from a transient dominant-negative enzyme isoform with a repressive neural activity into an enzyme which positively contributes to neural morphogenesis and maturation [40]. Therefore, it would be interesting to reveal whether similar alternative splicing-mediated switches also operate in other tissues where H3K9 demethylase activity has been reported.

LSD1 has also been identified to interact with many other proteins, some of them with a reported function in ESC biology (Table 1), others yet to be fully characterized, therefore expanding the plethora of cellular processes that can potentially be influenced by LSD1. 

## 4. Non-Canonical Targets of LSD1 beyond Demethylation of Histone Lysine Residues

Although LSD1 was originally identified as histone lysine demethylase, several reports have identified non-histone proteins as substrates of LSD1 [62]. In general, LSD1-mediated demethylation alters both the function and the stability of the target protein. Such substrates include proteins with important implications in stem cell biology (Table 2) being DNMT1 and OCT4 amongst them which will be further discussed in this review [10,63].

Catalytic-independent functions of LSD1 have also been reported, although its mechanistic understanding is limited [62]. For example, PKCα-dependent phosphorylation of LSD1 facilitated the recruitment of the circadian factors CLOCK: BMAL1 to the target promoters independently of LSD1 enzymatic activity [89]. Intriguingly, LSD1 promoted the proliferation of acute myeloid leukemia in the presence of the LSD1 inhibitor [90]. Additionally, LSD1 has been shown to interact with the tumor suppressor FBXW7 promoting its destabilization [91]. Specifically, binding to LSD1 impedes FBXW7 dimerization and leads to self-ubiquitylation of the FBXW7 monomer, followed by the rapid degradation through both proteasome and p62-mediated autophagy pathways [91]. Similarly, the interaction of LSD1 with p62 promoted the ubiquitylation of p62 followed by proteasomal degradation in a demethylation-independent manner [92]. On the contrary, the interaction of LSD1 with ERRα protected ERRα from proteasome-dependent degradation, thereby stabilizing the orphan nuclear receptor [93]. Similar demethylase-independent functions of LSD1 have been reported in zebrafish primitive hematopoiesis suggesting that this mechanism is likely to be evolutionarily conserved [94]. Therefore, it will be imperative to understand whether scaffolding activities of LSD1 also function during development.

## 5. LSD1: Self-Renewal or Pluripotency?

ESCs derived from the inner cell mass (ICM) of the developing blastocyst are pluripotent cells due to their properties of indefinite self-renewal in vitro and their ability to differentiate into all cell lineages of the three embryonic germ layers i.e., mesoderm, ectoderm and endoderm. The pluripotent state is primarily controlled by the core transcription factors *Oct4*, *Sox2*, and *Nanog*, which function together to regulate their own expression in an autoregulatory feedback loop. Additionally, the core transcription factors function activating and repressing the expression of pluripotency and lineage-specific genes, respectively. Upon a differentiation stimulus, multiple pathways orchestrate the loss of the core pluripotency factors through transcriptional and post-transcriptional mechanisms, leading the molecular switch from self-renewal to differentiation. Epigenetic modifiers have been shown to regulate self-renewal and differentiation by interacting with the core transcription factor circuitry.

**Figure 3:**
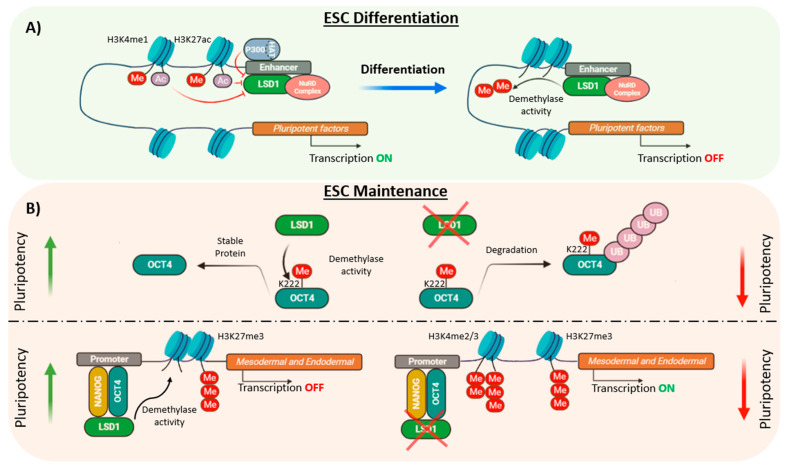
Role of LSD1 in ESC differentiation and maintenance. (**A**) LSD1 is not essential for ESC self-renewal but for differentiation. LSD1 is poised at the enhancers of pluripotent factors where p300/HAT inhibits its activity and therefore, transcription can occur. During differentiation, H3K4me1 LSD1-mediated demethylation switches off the expression of pluripotent factors. (**B**) LSD1 can affect pluripotency levels by demethylating OCT4 at residue K222, which impedes its degradation by the proteasome. In human ESCs, LSD1 occupies, together with OCT4 and NANOG, the promoters of mesodermal and endodermal genes. It demethylates H3K4me2/3 maintaining the silencing of developmental genes.

Several studies have pointed out the critical function of LSD1 during development as *Lsd1*-deficient mouse embryos die prior to gastrulation at E7.5 [10,26,95]. However, the impact of LSD1 in ESC self-renewal and pluripotency is controversial as it has been reported to be both dispensable and required for the maintenance of the ESCs characteristics. For instance, although *Lsd1* knockout mouse ESCs showed a severe growth impairment, these cells maintained the undifferentiated state, assessed by the cellular morphology and the expression of the pluripotency factors *Nanog* and *Oct4* [10]. Similarly, Foster et al., generated conditional *Lsd1* knockout mouse ESCs and observed a decrease in CoREST expression and its associated HDAC activity which did not affect ESC identity [96]. However, these cells displayed a differentiation impairment produced by the early overexpression of the mesodermal marker *Brachyury* together with the aberrant expression of other developmental markers such as *Hoxb7*, *Hoxd8* and the retinoic acid receptor γ (RARγ) [96]. Similarly, Whyte et al. reported that LSD1 is not required for ESC self-renewal but for differentiation [6]. Hence, by using chromatin immunoprecipitation coupled with massively parallel DNA sequencing (ChIP-seq) it was shown that LSD1, together with the NuRD complex, occupied enhancers of highly transcribed genes involved in pluripotency such as *Oct4*, *Sox2* and *Nanog*. Such enhancers were also bound by HAT p300 and nucleosomes with acetylated histones and therefore, LSD1 demethylase activity on histone substrates was inhibited by histone acetylation. However, upon differentiation, when the levels of p300 are decreased, the LSD1-NuRD complex demethylated H3K4me1, silencing ESC enhancers, a requirement to exit the ESC state and promote lineage specification (Figure 3A) [6]. In contrast, other studies have shown the role of LSD1 in ESC maintenance, both at post-transcriptional and at epigenetic level. Hence, it has been shown that LSD1 promoted OCT4 protein stability in pluripotent stem cells (PSCs) by demethylating OCT4 at the residue K222. Un-methylated OCT4-K222 was prevented from proteasome-independent degradation and thereby, LSD1-mediated demethylation of OCT4 promoted the transcription of PORE-motif genes preserving PSCs pluripotency (Figure 3B) [63]. In addition, LSD1 was reported to be critical for the maintenance of human ESCs through the silencing of developmental genes regulated by H3K4me2/3 and H3K27me3 markers by positioning in enhancers co-occupied by OCT4 and NANOG [97]. Thus, depletion of LSD1 in human ESCs was associated with a prompt expression of endodermal and mesodermal lineage markers, including *EOMES*, *BMP2*, *FOXA2* and *SOX17*, among others, due to an increase of H3K4me2/3 methylation levels leading to decreased cell growth and cellular differentiation (Figure 3B) [97]. The differences between this and other studies could be due to the use of ESCs from distinct species or could reflect the distinct pluripotent states, naive and primed, that mouse and human ESCs, respectively, represent [98].

## 6. LSD1 in Somatic Cell Reprogramming

In 2006, Takahashi and Yamanaka revolutionized stem cell research by reprogramming somatic cells into induced pluripotent stem cells (iPSC) by overexpressing the transcription factors *Oct4*, *Sox2*, *Klf4* and *c-Myc* (OSKM; generally referred as the Yamanaka factors) [99]. iPSCs resemble ESCs, having a similar developmental potential and being capable of contributing to the three germ layers [100]. Reprogramming to pluripotency is a complex process initiated by the downregulation of somatic transcriptional programs, followed by a mesenchymal-to-epithelial transition, and the expression of the core pluripotency factors. Notably, those changes in the expression of transcription factors require the transition from a somatic cell epigenetic status into an ESC-like state. Hence not surprisingly, accumulating evidence indicates that histone demethylases, including LSD1, might also have a role in the reprogramming efficiency.

**Figure 4:**
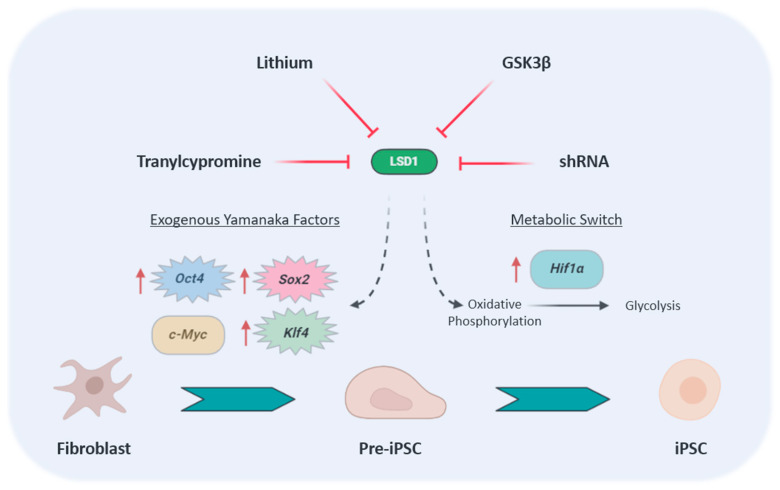
Function of LSD1 in reprogramming. LSD1 depletion by tranylcypromine, lithium, GSK3β or shRNA promotes iPSC reprogramming by two different pathways. Firstly, LSD1 inhibition enhances the exogenous expression of the Yamanaka factors, stimulating the conversion of fibroblast to pre-iPSC. Secondly, LSD1 inhibition leads to a metabolic switch from oxidative phosphorylation to glycolysis by rescuing *Hif1α* expression which promotes the conversion from pre-iPSC to iPSC [108].

Chemical compound-based direct reprogramming towards pluripotency offers a novel approach to generating iPSCs without viral vector-based genetic manipulation. Controversial studies have reported both positive and negative effects in the generation of iPSCs upon chemical inhibition of LSD1. It has been shown that inhibitor of LSD1 with tranylcypromine (also named parnate), in combination with inhibition of glycogen synthase kinase 3 (GSK3), enhanced reprogramming of human primary keratinocyte transduced with only two factors, namely *Oct4* and *Klf4* [101]. Similarly, iPSCs generation with one (*Oct4*) or two factors (*Oct4* in combination with either *Sox2* or *Klf4*) has been shown to be facilitated by the addition of Lithium (Li). The underlying mechanism also involves the inhibition of GSK3β, which in turn enhances the expression and transcriptional activity of *Nanog*, and the downregulation of LSD1 through a mechanism that remains unclear [102]. Moreover, Wang et al. showed that LSD1 silencing partially reproduced the Li effect in enhancing reprogramming, and that both combination of silencing LSD1 and Li treatment led to an additive effect on reprogramming efficiency in early stage, suggesting that LSD1 is a critical modulator for iPSCs generation [102]. Furthermore, the LSD1 inhibitor tranylcypromine, in combination with other small-molecule compounds, was sufficient to enable generation of iPSCs with a single transcription factor, *Oct4* [103]. The same authors improved the chemical cocktail and were able to generate iPSCs from mouse somatic cells with seven small-molecule compounds replacing the four Yamanaka factors [104]. Beneficial effects of LSD1 inhibition on reprogramming were also tested on hTERT-stabilized fibroblasts [105]. Thus, treatment with tranylcypromine or with a similar potent analog (Histone Lysine Demethylase Inhibitor RN-1) [106], activated an epithelial program which drives mesenchymal to epithelial transition (MET) [105], an essential process toward induced pluripotency [101] (Figure 4). To the best of our knowledge, we found only one report describing a negative effect on reprogramming upon silencing of LSD1 [107]. Wang et al., described that individual knockdown of the NuRD complex, including LSD1, increased mTOR transcriptional activation abrogating Sox2-mediated autophagy and inhibiting the reprogramming to pluripotency [107].

The mechanism behind the negative role of LSD1 in reprogramming has been recently elucidated [108]. Inhibition of LSD1 by tranylcypromine or by shRNA facilitated reprogramming at an early stage by both transcriptional and metabolic regulation. Hence, LSD1 inhibition promoted an accumulation of H3K4me1 levels which resulted in overexpression of the Yamanaka factors *Oct4*, *Sox2* and *Klf4* [108]. In addition, LSD1 inhibition facilitated a metabolic switch from oxidative phosphorylation to glycolysis which promoted the conversion from pre-iPSCs to iPSCs [109,110]. This change was in part produced by rescuing the expression of *Hif1α* which is normally reduced by the H3K4 demethylation activity of LSD1 [108] (Figure 4). Therefore, most of the studies show that LSD1 constitutes a barrier for efficient reprogramming.

## 7. Crosstalk with DNA Methylation

The eukaryotic genome is tagged by DNA methylation occurring predominantly at the C5 position of cytosine residues in the context of CpG dinucleotides, leading to the formation of 5-methylcytosine (5mC). This epigenetic mark plays a critical role in transcriptional silencing, heterochromatin formation, X chromosome inactivation, imprinting and genome stability, to name some examples [111]. Extensive DNA methylation is associated with the progression from a naive stem cell state into a more differentiated one [112]. De novo DNA methylation is established by the methyltransferases DNMT3A and DNMT3B, and the cofactor DNMT3L, which is catalytically inactive. DNA methylation is maintained through DNMT1 that recognizes hemi-methylated DNA and after replication, restores the fully methylation pattern from parent to daughter strand [113]. The roles of the three active DNMTs are different between mice and human ESCs. Hence, depletion of all mouse DNMT genes does not affect cellular viability as long as they are maintained in the undifferentiated state [114,115]. However, loss of DNMT1 in human ESCs results in global demethylation and death, whereas DNMT3A and DNMT3B knockout human ESCs are viable, with mild decrease of DNA methylation, and the potential to differentiate into the three germ layers [66]. DNA methylation and histone methylation are intimately connected to regulate chromatin structure and gene expression. For instance, DNMT1 is associated with the E3 ubiquitin protein ligase UHRF1 to maintain DNA methylation levels. During S phase, UHRF1 is recruited to hemi-methylated sites which are marked with H3K9me2/3, and it in turn ubiquitinates histone H3. Thereby, DNMT1 is recruited to replication forks by interaction with UHRF1, PCNA and ubiquitinated H3 [116,117,118].

**Figure 5:**
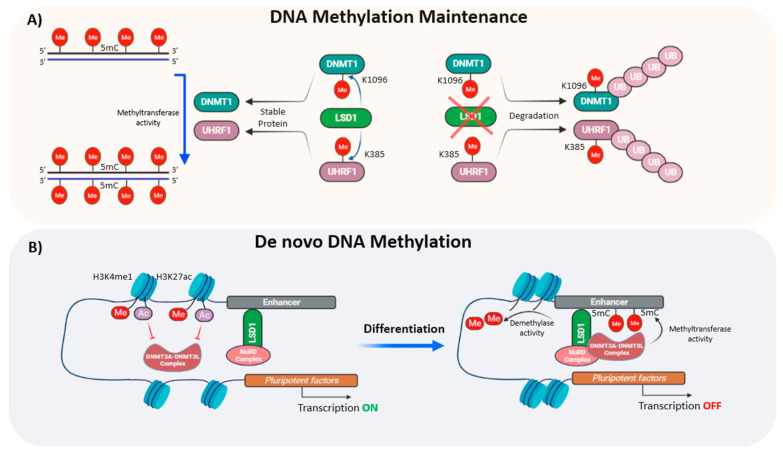
Crosstalk of LSD1 with DNA methylation. (**A**) DNMT1 and UHRF1 are methyltransferases involved in the maintenance of DNA methylation. LSD1 can demethylate DNMT1 and UHRF1 at the positions K1096 and K385, respectively, preventing proteasome-mediated degradation and subsequent hypomethylation of the DNA. (**B**) LSD1-mediated demethylation of H3K4me1 at pluripotent enhancers promotes the deposition of 5mC by DNMT3A-DNMT3L, repressing the expression of pluripotency factors during differentiation. In stem cells, H3K27ac inhibits the methyltransferase activity of the DNMT3A-DNMT3L complex.

DNMT1 is a substrate of LSD1 (Table 2). It has been reported that LSD1-mediated demethylation of DNMT1 at the K1096 residue prevents its degradation by the proteasome, and in turn, stabilizes the protein. Thus, loss of LSD1 in mouse ESCs leads to a progressive depletion of global DNA methylation at both unique and repetitive sequences, suggesting that LSD1 plays a central role in maintaining, rather than establishing, DNA methylation (Figure 5A) [10]. However, a later study showed that UHRF1 is also subjected to methylation-mediated protein degradation (Table 2), and that LSD1 regulates UHRF1 protein stability in cancer cells [87]. Since DNA methylation is more sensitive to depletion of UHRF1 than that of DNMT1 and moderate reduction of UHRF1 but not DNMT1 can lead to a reduction of global DNA methylation (Figure 5A) [119], Zhang et al., concluded that LSD1 was more likely to control this epigenetic mark though UHRF1, rather than DNMT1, stabilization. It would be interesting to understand whether similar mechanisms involving the UHRF1-LSD1 axis also function in pluripotency and self-renewal of ESCs.

An additional mechanism by which LSD1 might influence DNA methylation includes its histone demethylase activity. Hence, it has been well-established that H3K4 methylation protects gene promoters from recruitment of the DNMT3A-DNMT3L complex [120], suggesting that LSD1 facilitates the access of de novo methyltransferases. In agreement with this observation, it has been reported that demethylation of H3K4me1 at enhancers of pluripotency genes facilitates the binding of DNMT3A through interaction with the LSD1-Mi2/NuRD complex, leading to complete silencing of pluripotency genes during ESC differentiation (Figure 5B). Importantly, this mechanism is specific to pluripotency enhancers, as DNA methylation of repetitive elements containing the H3K4me0 mark is maintained independently of LSD1 activity [121].

## 8. Perspectives

PSCs, including ESCs and iPSCs, recapitulate many aspects of in vivo pluripotency. Therefore, they represent a good model to study development where the precise spatiotemporal regulation of gene expression is critical to ensure proper lineage commitment, cell fate determination, and ultimately, organogenesis. While much work remains to be carried out, our understanding of the mechanisms that govern self-renewal and pluripotency has dramatically increased during the past years. Accumulating evidence has suggested that LSD1 maintains epigenetic signatures, through the regulation of histone methylation and likely DNA methylation, that are fundamental to maintain ESC identity and for the activation or repression of genes during ESCs differentiation. In addition, the development of specific inhibitors of LSD1 has guided progress toward efficient somatic cell reprogramming. However, there are important discoveries that are yet to be made.

LSD1 is part of distinct protein complexes whose epigenetic function in inhibiting or enhancing transcriptional programs is likely context-dependent or even species-specific. In order to address the role of LSD1 in ESC biology, a more defined understanding of the biochemical nature of LSD1-containing protein complexes is required, coupled with a dissection of how these activities function in enhancers and promoters of the different target genes. Noteworthy, the discovery of non-histone substrates expands the plethora of cellular processes regulated by LSD1. We believe that with the development of high-throughput approaches to identify and characterize methylated proteins, the number of substrates of LSD1 will increase. An important task will be to further elucidate the LSD1-mediated demethylation effects on these non-histone substrates. Moreover, noncatalytic targets of LSD1 have also been identified, adding an extra layer of complexity to the roles of LSD1. This non-canonical activity of LSD1 is becoming increasingly relevant in cancer, the biological clock and hematopoiesis, and we expect that additional examples in ESC biology will soon be elucidated. Clearly, the extent to which these non-histone substrates regulate self-renewal and pluripotency remains to be investigated, but they may indeed be the norm rather than the exception.

Last but not least, the combined efforts of multiple laboratories have helped to elucidate the interplay amongst distinct epigenetic marks. While much work remains to be carried out, our understanding of how DNA methylation and histone modifications are closely interconnected to control gene expression is becoming more evident. Yet, the central role of LSD1 in DNA methylation is poorly understood and many questions remain to be answered. Does LSD1 control methylation through targeting DNMT1, UHRF1 or through its histone demethylase activity? Does LSD1 influence global 5mC deposition or is it specific to pluripotency enhancers? In many cases, these mechanisms appear to be context-specific, stressing those additional efforts are needed to elucidate the crosstalk of LSD1 and DNA methylation.

We are hopeful that more integrative approaches will reveal the complexity of LSD1 regulation in ESCs. This knowledge will not only advance our understanding in development but also in regenerative medicine. The discovery of selective inhibitors targeting different LSD1 domains will advance our understanding of both its canonical and non-canonical function in ESC biology. Moreover, given the association between high levels of LSD1 and malignant neoplasia, such understanding will surely improve cancer therapies. We believe that the many faces of this multifaceted and fascinating protein will be fully appreciated in the near future.

## Figures and Tables

**Table 1:** Protein interactors of LSD1. Only interactors of LSD1 with a reported function in ESCs are shown.

Interactor	Function in ESCs	References
RCOR2	RCOR2 regulates pluripotency via suppressing lineage-specific genes and the reprogramming of somatic cells to iPSCs.	[41,42]
HDAC1	HDAC1/2 induce the transcriptional program of self-renewal-associated genes such as *Oct4*, *Nanog*, *Esrrb*, and *Rex1*, thereby regulates the pluripotency of ESCs.	[43]
ZNF217	ZNF217 has a critical role in ESC self-renewal by restricting the METTL3 methyltransferase activity.	[44]
MTA	MTA2 and MTA3, but not MTA1, preserve human ESCs from differentiating into the mesodermal lineage.	[45]
RBBP4 and 9	RBBP4 and 9 regulate ESC self-renewal by sustaining the transcription of core pluripotency factors and inhibiting the genes involved in organogenesis.	[46,47,48]
MBD2	Two isoforms of MBD2, MBD2a and MBD2c, with contrasting roles: MBD2a enhances ESC differentiation through recruitment of the NuRD complex while MBD2c facilitates reprogramming. Mbd2/NuRD is also essential to maintain normal chromatin structure and gene regulation in ESCs.	[49,50]
MBD3	MBD3 is a scaffolding protein essential for NuRD complex assembly. MBD3/NuRD hinders the expression of pluripotency and preimplantation transcripts allowing cells to exit self-renewal for proper lineage-commitment. It is important to maintain normal chromatin structure and gene regulation in ESCs. Conflictive data in enhancing and suppressing reprogramming.	[35,50,51,52,53,54,55]
CHD4	CHD4 suppresses the aberrant expression of *Tbx3*, which mainly impairs endoderm differentiation.	[56]
ZMYM2	ZMYM2 plays a central role in transcriptional regulation of ESCs. It represses the expression of *NANOG* and *OCT4* during early differentiation allowing ESCs to exit from the pluripotency state.	[57]
CTBP1	CTBP1/2 is a core regulator of PRDM14-mediated transcriptional repression which is a prerequisite for transition from primed to the naïve state.	[58]
MLL1	MLL1-mediated H3K4me1 deposition at enhancers regulates cell-fate determination and its blockage reinforces naïve reprogramming.	[59]
Snail1	Snail1 is dispensable for ESC self-renewal, however, it steers EpiSC exit and modulates neuroectodermal, endodermal and mesodermal specification. It also enhances reprogramming.	[60,61]

**Table 2:** Non-histone substrates of LSD1. Name of the substrate, the effect of Lys demethylation and the role (if any) in ESCs are depicted.

Substrate	K Position	Effect	Role in ESCs	References
E2F1	185	Stabilization of E2F1 and activation of proapoptotic genes.	N/A	[64,65]
DNMT1	1096 and 142	Removal of the methyl group from K1096 (mouse), K1094 (human), and K142 of DNMT1 increases stability. K142 demethylation in the S-phase promotes stability by restricting L3MBTL3-CRL4^DCAF5^-mediated proteolysis.	DNMT1 is essential for ESCs cell viability and surveillance by controlling DNA methylation.	[10,66]
p53	370	Inhibition of the transcriptional activity of p53.	Upon DNA damage, activated p53 represses the core ESC transcriptome and induces the expression of lineage-specific markers. p53 is a transcriptional regulator which suppresses *Nanog* expression during ESCs differentiation.	[9,67]
MEF2D	267	Enhances its transcriptional activity.	Promotes myogenic differentiation.	[68]
ERa	266	Demethylation of K266 allows subsequent acetylation leading to activating of ERα target genes.	N/A	[69]
HSP90	615	It promotes HSP90 degradation.	It regulates pluripotency by: (i) regulating OCT4, NANOG and pSTAT3 expression and prevention of proteasomal-mediated degradation of OCT4 and NANOG; (ii) modulating *Oct4* mRNA, particularly restraining ESC from mesoderm differentiation.	[70,71]
AGO2	726	Stabilization	Its expression promotes an accelerated differentiation by increasing let-7 microRNAs which inhibits Trim71 translation.	[72,73]
HIF-1a	391	Demethylation of HIF1α at K391 prevents proteasomal-mediated degradation and PHD2-induced hydroxylation, thereby enhancing transcriptional activity of HIF1α to facilitate VEGF expression.	Activated HIF1α enhances the glycolytic program leading to efficient reprogramming. It also sustains self-renewal of iPSCs through regulating Actl6a and acetylation. Inhibition of HIF1α promotes endoderm and mesoderm differentiation.	[74,75]
MTA1	532	K532 demethylation disorganizes the formation of the NuRD repressor complex. Unmethylated MTA1 promotes acetylation of demethylated histone H3K9 shifting gene repression to activation.	MTA1 forms a complex with NANOG and POU5F1 known as a NODE. MTA1 deficiency upregulates the expression of endoderm-associated markers.	[76,77]
STAT3	140	K140 demethylation enhances transcriptional activity in response to IL-6.	STAT 3 controls *Myc* expression, promoting self-renewal and pluripotency in ESCs. Its activation is essential for the reprogramming of terminally differentiated cells.	[78,79,80]
MYPT1	442	K442 demethylation destabilizes MYPT1 and increases RB1 phosphorylation leading to cell cycle progression.	N/A	[81]
OCT4	222	Prevents proteasome independent degradation and refrains the ‘locked-in’ mode binding of OCT4 homodimers which enhances the expression of target genes.	OCT4 is a core pluripotency factor.	[82,83,84,85]
UHRF1	385	K385 demethylation stabilizes UHRF1.	It associates with Setd1a/COMPASS complex to maintain mesoderm and neuroectoderm histone marks, ensuring a proper differentiation in stem cells. In association with the Setd1a/COMPASS complex, UHRF1 aids in the regulation of H3K4me3 and H3K27me3 methylation. The maintenance of bivalent histone marks ensures efficient mesoderm and ectoderm differentiation.	[86,87,88]
